# Immunoglobulin Classification Based on FC* and GC* Features

**DOI:** 10.3389/fgene.2021.827161

**Published:** 2022-01-24

**Authors:** Hao Wan, Jina Zhang, Yijie Ding, Hetian Wang, Geng Tian

**Affiliations:** ^1^ Institute of Advanced Cross-field Science, College of Life Science, Qingdao University, Qingdao, China; ^2^ Geneis (Beijing) Co., Ltd., Beijing, China; ^3^ Yangtze Delta Region Institute (Quzhou), University of Electronic Science and Technology of China, Quzhou, China; ^4^ Beidahuang Industry Group General Hospital, Harbin, China

**Keywords:** immunoglobulin classification, machine learning, key feature extraction, MRMD, autoprop

## Abstract

Immunoglobulins have a pivotal role in disease regulation. Therefore, it is vital to accurately identify immunoglobulins to develop new drugs and research related diseases. Compared with utilizing high-dimension features to identify immunoglobulins, this research aimed to examine a method to classify immunoglobulins and non-immunoglobulins using two features, FC* and GC*. Classification of 228 samples (109 immunoglobulin samples and 119 non-immunoglobulin samples) revealed that the overall accuracy was 80.7% in 10-fold cross-validation using the J48 classifier implemented in Weka software. The FC* feature identified in this study was found in the immunoglobulin subtype domain, which demonstrated that this extracted feature could represent functional and structural properties of immunoglobulins for forecasting.

## 1 Introduction

Immunoglobulins, or antibodies, are a group of proteins secreted by B lymphocytes that recognize invading antigens and bind to antigens with high affinity and specificity to neutralize toxic substances. In general, antibodies are composed of two identical polypeptide chains, each with a light chain and a heavy chain (Narciso et al., 2011). They can be divided functionally into variable (V) domains, which bind to antigens, and constant (C) domains, which activate, complement, or bind to Fc receptors (Schroeder and Cavacini, 2010). To predict the structure of immunoglobulins, (Lepore et al., 2017) developed the PIGSPro Server, an updated version of the popular PIGS Server.

Immunoglobulins have a pivotal role in disease regulation. Therefore, human and nonhuman polyclonal immunoglobulins have been used in therapeutics for many years. Five monoclonal immunoglobulins ranked in the top 10 blockbuster biotherapeutics drugs (Norman et al., 2020). Patients with primary immune deficiencies greatly benefit from the intravenous or subcutaneous administration of human immunoglobulin preparations (Perez et al., 2017). The advanced development of medicine is urged by its finite supply, which requires more identification of valuable therapeutic immunoglobulins. However, biochemical experiments are time-consuming with enzymes to fragment immunoglobulin molecules (Schroeder and Cavacini, 2010) or X-ray crystallography to obtain accurate structures (Narciso et al., 2011).

Machine learning can identify desired proteins from a large number of sequences within a short time to guide the experimental discovery process (Guo et al., 2020; Liu et al., 2020; Song G. et al., 2021; Cheng et al., 2021; Deng et al., 2021; Dong et al., 2021; Guo et al., 2021; Tang et al., 2021; Yu et al., 2021; Zhao et al., 2021). Over the past decades, researchers have developed many machine learning–based techniques for protein sequence analysis (Zhai et al., 2020; Zeng et al., 2020; Chen et al., 2021; Li et al., 2021). The bioinformatics approach of identifying immunoglobulins is to convert protein sequences into numerical vectors to reveal the internal structures of proteins. The critical aspects of protein identification can be listed as follows: feature extraction, feature selection, and machine learning. Feature extraction methods include *n-gram* feature type: amino acid composition (AAC), Dipeptides (Dip), Tripeptides, where frequencies of *n*-length peptides are used as feature vectors (Ding et al., 2011; Gautam et al., 2013; Diener et al., 2016; Rahman et al., 2018; Liu et al., 2019; Lv et al., 2019; Fu et al., 2020; Wang H. et al., 2021; Wang J. et al., 2021; Zhai et al., 2020; Shao and Liu, 2021; Yang et al., 2021; Zhang et al., 2021). In addition, pseudo–amino acid composition (PseAAC) is also a widely adopted feature extraction method, including physicochemical properties between residues (Hansen et al., 2008; Sanders et al., 2011; Gautam et al., 2013; Chen et al., 2016; Diener et al., 2016; Khan et al., 2020; Awais et al., 2021; Naseer et al., 2021).

Many feature types and complex classification methods may generate redundant information (Song B. et al., 2021). Therefore, some studies began to eliminate redundant parts to improve the predictive performance of classification models. This process is also called feature selection. MRMD (Zou et al., 2016; Ao et al., 2020; Li et al., 2020a; Li et al., 2020b; Meng et al., 2020) and ANOVA (Anderson, 2001; Lv et al., 2019) are standard feature selection methods. For optimal feature identification, (Feng et al., 2021) uses the PCA and MCE methods to make the features orthogonal and obtain the core feature set with the minimum 10-dimensional attributes for PPR gene identification and realized 97.9% accuracy. (Li et al., 2020b) used a 19-dimensional feature model to classify anticancer peptide sequences. (Ao et al., 2020) used a 10-dimensional feature model to classify antioxidant proteins and realized 90.44% accuracy. (Meng et al., 2020) used a 6-dimensional feature model to classify cell wall lytic enzymes.

However, very few tools have been developed for immunoglobulin identification. (Tang et al., 2016) used the pseudo amino acid composition (PseAAC) feature extraction approach to realize over 96% prediction accuracy in their pioneering work on immunoglobulin identification. (Gong et al., 2021) used the CC–PSSM and monoTriKGap feature extraction, MRMD feature selection, and single dimension reduction methods to realize 92.1% immunoglobulin identification accuracy by two-dimensional features. However, the link between optimal features and functional structures of immunoglobulins remains to be investigated.

**FIGURE 1:**
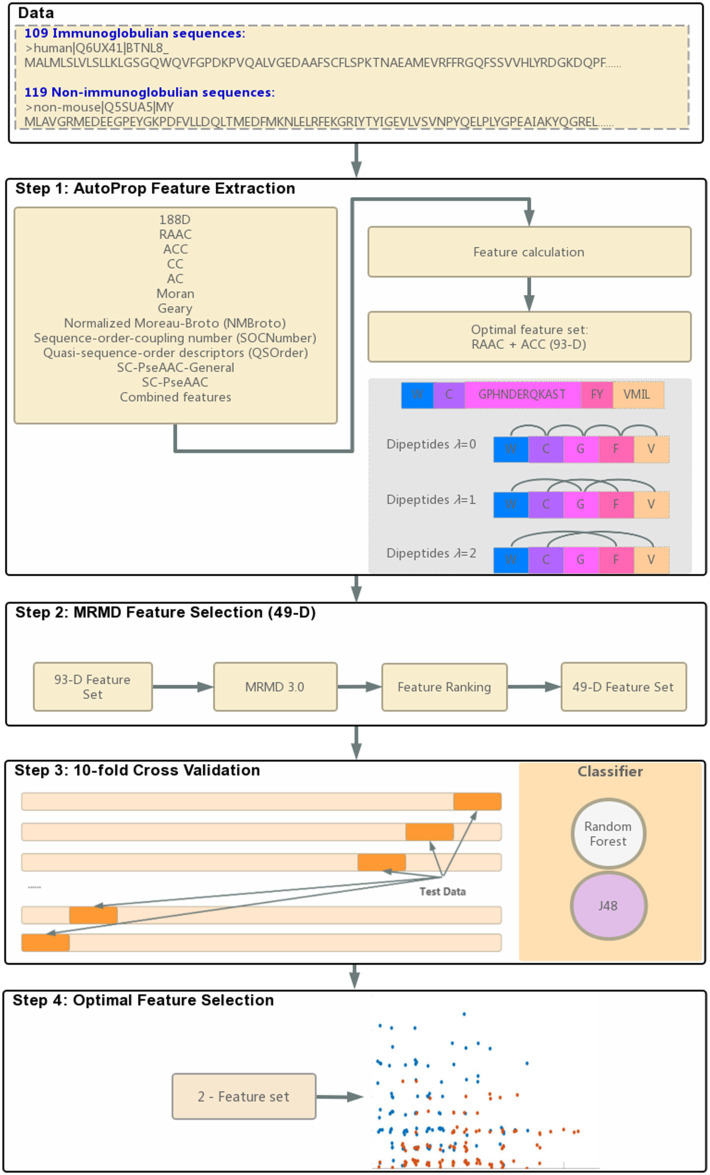
Flowchart of identifying immunoglobulins.

To obtain a diverse feature set, this study integrated 188-D physicochemical properties, auto-cross covariance (ACC) information, and dipeptide compositions of reduced amino acids. Dimensions were reduced using the max-relevance-max-distance (MRMD) method and the single dimension reduction method. The RF and J48 classifiers implemented in Weka software were used to identify immunoglobulins. Finally, two features can correctly predict immunoglobulins, FC* and GC*. The entire modeling process is illustrated in Figure 1. The FC* feature identified in this study was found in immunoglobulin subtype domain IPR003599, which demonstrated that this extracted feature could represent functional and structural properties of immunoglobulins for forecasting.

## 2 Materials and Methods

### 2.1 Datasets

Data for this study were collected by (Tang et al., 2016), which contain 228 samples (109 immunoglobulin samples and 119 non-immunoglobulin samples) extracted from the Universal Protein Resource (UniProt).

### 2.2 RAAC

Polypeptide chains fold to tertiary structures based on the physicochemical properties of residues (Tang et al., 2016). Analyzing the occurrence frequency of residue compositions cannot visualize three-dimensional protein structures. The reduced amino acid cluster (RAAC) method, replacing protein sequences with less than 20 amino acid alphabets based on a specific reducing scheme, can reduce sequence complexity. With removing non-essential information, functionally conserved regions will be displayed more clearly. Recent work presented 3D protein structures of ectonucleotide pyrophosphatase with a 1D view using the RAAC method (Solis, 2015; Zheng et al., 2019).

There are many choices of reduced schemes, and different decisions could produce distinctive protein classification results. For example, the RAACBook web server provided 74 types of reduced amino acid alphabets derived from over 1,000 published articles in PubMed (Zheng et al., 2019). Bins within the scheme are related to the chemical properties of amino acids. Dayhoff classes (AGPST, DENQ, HKR, ILMV, FWY, and C) are most used. Also, S and T are frequently together, and so are K and R, D, and E (Susko and Roger, 2007).

We used the AutoProp (Feng et al., 2020) to screen out the optimal reduced scheme of the immunoglobulin and non-immunoglobulin sequences. GPHNDERQKAST, FY, VMIL, C, and W (Figure 1 Step 1) were used. Under this reduced scheme, the 20 amino acid alphabets were represented by five alphabets: G, F, V, C, and W. For instance, any amino acid that is a G, P, H, N, D, E, R, Q, K, A, S, or T is then treated as character G. For any amino acid F and Y, it is then treated as character F, and so forth.

### 2.3 Feature Extraction

A sequence can be represented by sequential form and discrete form. Homolog sequences can be compared with the BLAST or FASTA program benchmark datasets for traditional sequence comparison methods. However, the similarity-based way is unsuitable for distantly related sequences (Wei et al., 2014; Chen et al., 2016; Jin et al., 2019; Manavalan et al., 2019; Hong et al., 2020; Tang et al., 2020; Wang et al., 2020; Ding et al., 2021a; Ding et al., 2021b; Huang et al., 2021; Shao et al., 2021). By converting amino acid codes to a series of discrete numerical vectors, the discrete form can overcome this drawback and be used by machine learning for protein classification. Sometimes, proteins can be classified according to fewer features, while BLAST cannot.

Different numerical values of protein codes mean different feature descriptors. Feature descriptors provided by AutoProp include 188D, ACC, PseAAC, and another nine methods (Figure 1 Step 1). Also, AutoProp provides combined features between those methods. The built-in classifiers will then calculate the accuracy percentage of each feature and decide the optimal feature.

For our data, the optimal feature is the combined features of RAAC and ACC. RAAC features also represent dipeptides of reduced amino acid, like CV, C*V (λ-gap = 1), and C**V (λ-gap = 2). The following formula was used to calculate the values of those features:

$$f_{u}=\frac{n_{u}^{\lambda }}{\sum n_{u}^{\lambda }},$$

where λ = 0,1,2, and $n_{u}^{\text{λ}}$ denotes the number of λ-gap dipeptides of type *u* in a protein sequence.

ACC means the autocross covariance (ACC) transformation and contains auto covariance (AC) and cross-covariance (CC) and is introduced to transform protein sequences into fixed-length vectors (Feng et al., 2020). With its ability to identify sequence homologies, ACC has been successfully used for protein family classification and protein interaction prediction (Dong et al., 2009).

### 2.4 MRMD

The main disadvantage of the sequence word frequency vector is that they are usually huge. Therefore, dimension reduction, also called feature selection, is chosen for protein classification. The MRMD method, which is the max-relevance-max-distance–based dimensionality reduction method, is more considered for relationships among features and stability of feature selection. Cross-validation and the ROC curve are usually used to evaluate classification accuracy. The MRMD method can reduce feature dimensions with few accuracy drops (Zou et al., 2016; He et al., 2020; Tao et al., 2020).

### 2.5 Performance Measurement

We used three metrics to evaluate model performance. Indicators include sensitivity (SE), specificity (SP), and Accuracy (Jiang et al., 2013; Wang X. et al., 2021). Calculation methods are described as follows:

$$\text{SE}=\;\frac{TP}{TP+FN}\text{,}$$

$$\text{SP}=\frac{TN}{TN+FP}\text{,}$$

$$\text{Accuracy}=\frac{TP+TN}{TP+FN+TN+NP}\text{,}$$

where TN, TP, FN, and FP refer to the numbers of correctly predicted non-immunoglobulin proteins, correctly predicted immunoglobulin proteins, incorrectly predicted non-immunoglobulin proteins, and incorrectly predicted immunoglobulin proteins, respectively. Sensitivity (SE) is also known as recall, and it measures the percentage that positive samples can be expected correctly over all the samples. SP indicators measure the probability of negative samples classified as non-immunoglobulins, and Accuracy is used to evaluate the overall performance of a prediction model.

## 3 Results and Discussion

### 3.1 Classification Results Under Different Features

**FIGURE 2:**
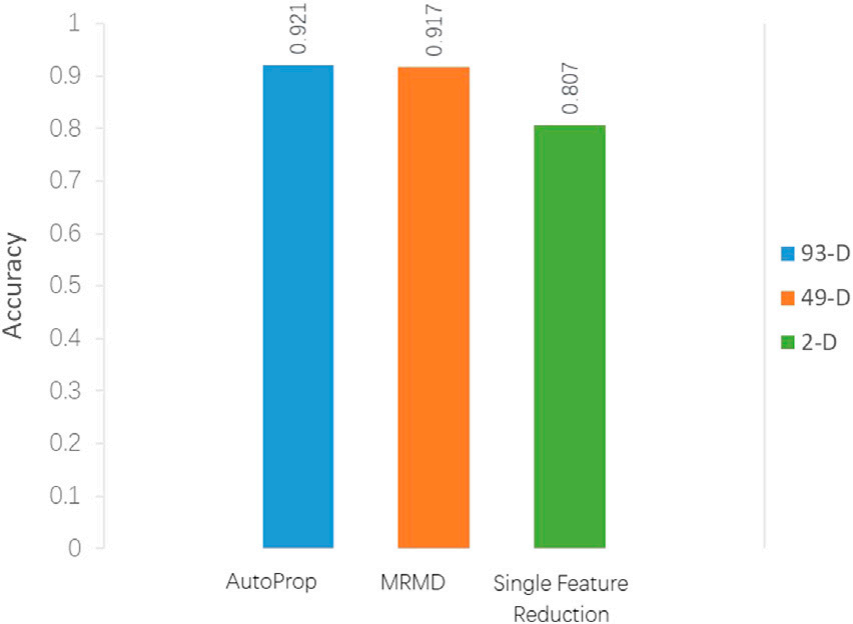
Classification accuracy comparison between models with different feature selection methods.

Props returned 93D best features, and the frequency of dipeptides (λ-gap = 0, 1, 2) is saved in features 1–75, followed by 18 ACC features. The classification accuracy was 92.1% in the RF classifier and 10-fold cross-validation using Weka software. The MRMD method further reduced the dimension to 49D, and accuracy was 91.7% using the same classifier. It can be seen that MRMD reduces nearly half of the feature dimension, but the accuracy is only dropped by 0.4% (Figure 2). After continuous attempts to reduce features, the optimal two features (GC* and FC*) are finally obtained; the classification accuracy was 80.3% using the J48 classifier in Weka.

### 3.2 2D Features Scatter Distribution

**FIGURE 3:**
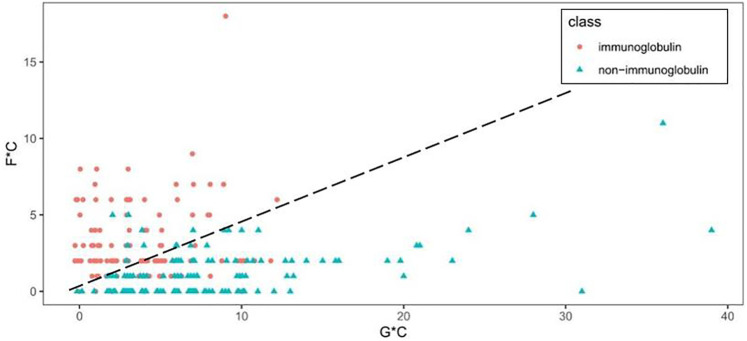
Scatter plot of GC* and FC* features.

Figure 3 shows the scatter plot of GC* and FC* features. What stands out in Figure 3 is that immunoglobulin and non-immunoglobulin samples can be distinguished. Immunoglobulins are scattered on the upper left with higher FC* values, and non-immunoglobulins are found in the lower right with higher GC* values. For 118 out of 119 non-immunoglobulin samples, the FC* value is equal to or less than 5. Among these, the FC* value of 49 samples is zero. The GC* value for immunoglobulin samples is less than or equal to 12.

### 3.3 Interpretation of Feature FC*

**FIGURE 4:**
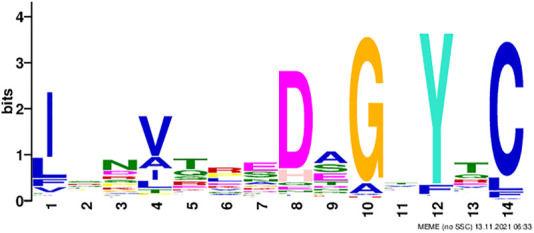
Motif discovered among immunoglobulin sequences using the MEME tool; the height of the letter indicates its relative frequency at the given position within the motif.

We noticed 49 out of 119 non-immunoglobulin samples had an FC* value of zero, whereas only four immunoglobulin samples had an FC* value of zero. Using motif search website MEME Suite 5.4.1 (Bailey and Elkan, 1994; Bailey et al., 2009) and running 109 immunoglobulin sequences, results showed that 107 out of 109 immunoglobulin samples had a motif, “ISNVTREDAGTYTC” (Figure 4). Based on the reduced scheme, Y was treated as F.

Immunoglobulin sequences were subjected to InterProScan (Zdobnov and Apweiler 2001) to understand the motif structure better to map protein domains. Results showed that the finding motif belonged to immunoglobulin subtype domain IPR003599.

**FIGURE 5:**
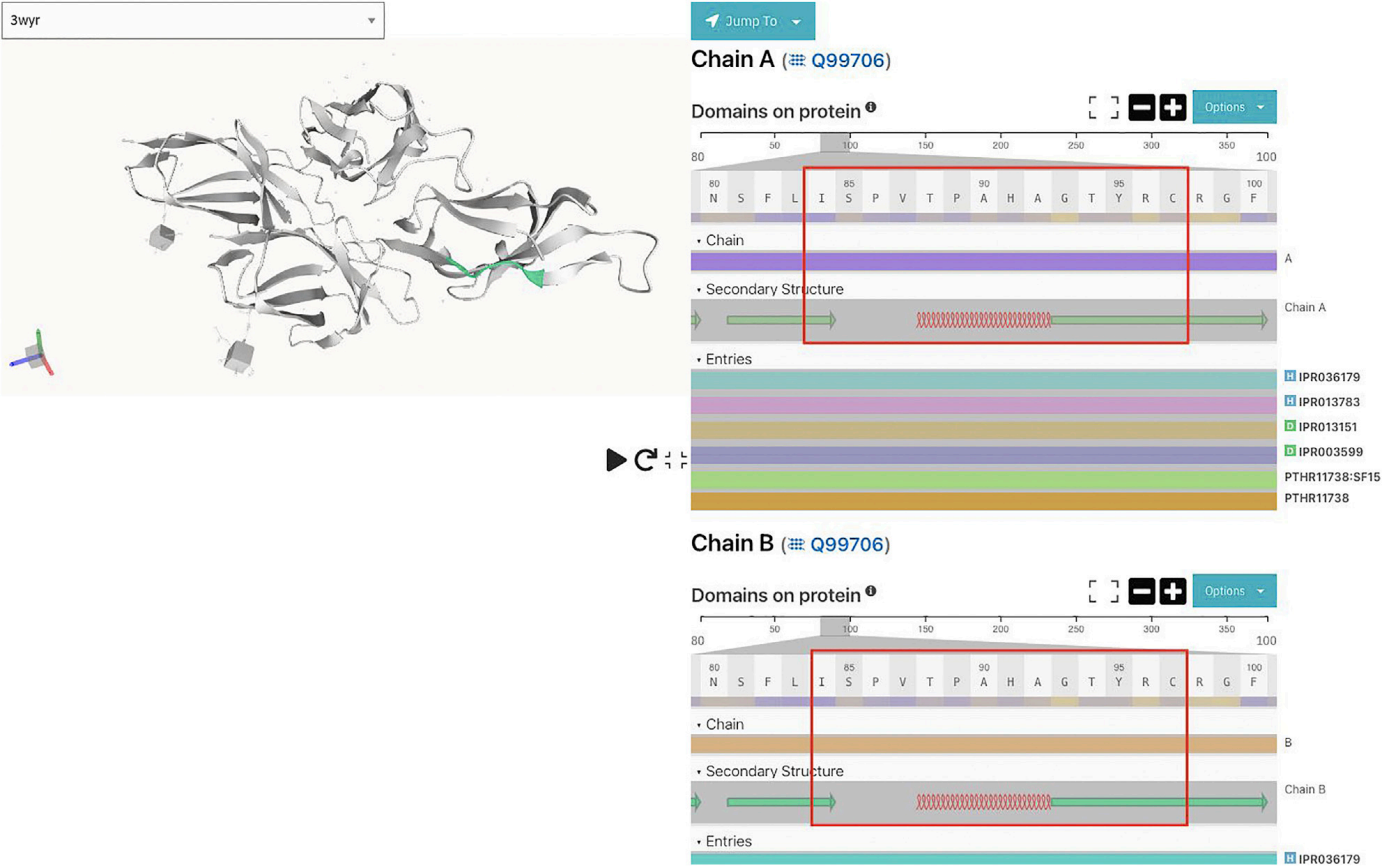
Shared motif and its secondary structure (from PDB entry 3wyr) using InterproScan.

Also, secondary structure predictions of the motif using JPred (Drozdetskiy et al., 2015) predict that the shared motif comprises alpha helices and beta sheets separated by disordered regions (Figure 5).

## 4 Conclusion

The present research aimed to examine a method to classify immunoglobulins and non-immunoglobulins using two features, GC* and FC*. Classification of 228 samples (109 immunoglobulin samples and 119 non-immunoglobulin samples) revealed that the overall accuracy was 80.7% in the J48 classifier and 10-fold cross-validation using Weka software. The FC* feature identified in this study was found in immunoglobulin subtype domain IPR003599, which demonstrated that this extracted feature could represent functional and structural properties of immunoglobulins for forecasting.

## Data Availability

Publicly available datasets were analyzed in this study. These data can be found here: DOI: 10.1039/c5mb00883b.
